# Dental Register, or a Record for Detailing Facts in Each Case of Dental Practice

**Published:** 1846-09

**Authors:** 


*Dental Register,* or a record for detailing facts in each case of dental practice. By H. Crane, D. D. S., of Cincinnati, Ohio.

The above is the title of a case book, which Dr. C. O. Cone has had the kindness to place in our hands for examination; and it is certainly one of the most complete and convenient things of the kind we have ever seen. Every dentist should keep a correct record of each case occurring in his practice, noting at the time its peculiarities, with such observations in relation thereto as his experience and knowledge of dental pathology may suggest; and we have seen no plan by which this could be so readily and accurately done, as that furnished by Dr. Crane's Register. In this, he will be able, in half a minute, to designate every operation which he may have performed, and this done, in a blank space provided for the purpose, he may append such remarks as may be suggested to his mind by the nature or peculiarity of the case.

Facts and observations, thus recorded, would be interesting and useful to every practitioner. By this means, a dentist might, at every future visit of his patient, refer to them, and ascertain, by the result of the operation or treatment which he had instituted, the correctness of the opinion which he had formed at the time in relation to the case. We have long been in the habit of keeping a record of the cases which have occurred in our own practice, and so much are we pleased with this Register, that we intend ordering one for our own use.—*Balt. Ed.*

